# Visual Strategies for Guiding Gaze Sequences and Attention in Yi Symbols: Eye-Tracking Insights

**DOI:** 10.3390/jemr18050057

**Published:** 2025-10-16

**Authors:** Bo Yuan, Sakol Teeravarunyou

**Affiliations:** School of Architecture and Design, King Mongkut’s University of Technology Thonburi, Bangkok 10140, Thailand; bo.yuan@kmutt.ac.th

**Keywords:** Yi graphics, eye-tracking, gaze sequence, attention, visual strategies

## Abstract

This study investigated the effectiveness of visual strategies in guiding gaze behavior and attention on Yi graphic symbols using eye-tracking. Four strategies, color brightness, layering, line guidance, and size variation, were tested with 34 Thai participants unfamiliar with Yi symbol meanings. Gaze sequence analysis, using Levenshtein distance and similarity ratio, showed that bright colors, layered arrangements, and connected lines enhanced alignment with intended gaze sequences, while size variation had minimal effect. Bright red symbols and lines captured faster initial fixations (Time to First Fixation, TTFF) on key Areas of Interest (AOIs), unlike layering and size. Lines reduced dwell time at sequence starts, promoting efficient progression, while larger symbols sustained longer attention, though inconsistently. Color and layering showed no consistent dwell time effects. These findings inform Yi graphic symbol design for effective cross-cultural visual communication.

## 1. Introduction

Yi graphic symbols, rooted in the traditional script and motifs of the Yi (Nuosu) ethnic group in southern China, embody a rich cultural heritage encompassing mythological, shamanistic, and ritualistic elements. These symbols, often depicting deities, natural forces, and ancestral spirits, serve as protective talismans and communicative tools conveying identity and spiritual significance [1]. In contemporary design, integrating Yi symbols into modern artistic styles has gained prominence, preserving cultural heritage while enhancing visual communication and cultural expression [2].

Optimizing Yi graphic symbols for effective communication is crucial, as they function as icons that enhance intuitive understanding and user engagement. Icons and symbols in design simplify complex information, improve accessibility, and establish visual hierarchies [3]. In the context of Yi graphic symbols, this requires analyzing gaze sequences and attention to ensure effective guidance, particularly in multimedia interfaces or packaging where cultural authenticity meets modern usability [4,5,6,7,8]. Eye-tracking studies highlight the role of visual elements like color and shape in regulating attention and improving communication efficiency [9].

Visual design research has extensively explored gaze sequences using eye-tracking, revealing patterns such as F-shaped or Z-shaped scanning behaviors in web and print layouts, where users prioritize hierarchical over linear scanning [10]. These patterns, rooted in left-to-right, top-to-bottom Western reading habits, reflect how users navigate alphabetic scripts and optimize page layouts. However, these patterns may not fully apply to Yi symbol reading, a logographic and syllabic script used by the Yi ethnic group in China. Although our current study does not focus on a text-based analysis, a previous experiment revealed that subjects tended to gaze at Yi symbols in a left-to-right sequence, mimicking a reading-like pattern despite the non-linear, logographic nature of the script [11]. This finding suggests that cultural or perceptual influences may impose a familiar scanning habit, even in the absence of textual linearity.

Designers use strategies like leading lines, size variations, and color brightness to manipulate gaze sequences, as demonstrated in empirical studies assessing composition effectiveness. For instance, Gayen et al. [9] claim that brighter colors capture attention more effectively than dull colors. The fixation time on yellow was found to be longer than on red. On a dark background, yellow stands out more strongly than red. A yellowish target tends to be more salient than a bluish one, through red and green show similar salience in those comparisons [12]. From the perspective of Yi culture, however, red has traditionally been used in products more frequently than yellow. This contrast makes it interesting to experiment further by comparing which color captures visual attention more effectively. They also claim that the larger graphic components and guiding lines significantly influenced gaze trajectories, with eye-tracking data showing reduced gaze sequence deviations when designs incorporated familiar shapes and perspectives. However, such studies often focus on general or Western designs, with limited application to ethnic symbols like Yi graphics.

A previous experiment on Yi graphic symbols [11] examined gaze sequences across eight image pairs designed in accordance with SendPoints’ layout principles [13]. Thirty participants completed eye-tracking tests, which indicated deviations from these guidelines. The guidelines represent a set of design heuristics for arranging visual elements to guide viewer attention, rather than fixation guidelines. Only one pair, characterized by a bottom-up flow with a photographic element, achieved 90% adherence. In this configuration, a photograph of a mountain was positioned at the bottom and a text logo at the top. The expansive mountain image effectively guided the viewer’s gaze upward toward the logo, suggesting that a larger visual element can direct attention to a smaller one. An open question remains, however: if the stimulus consists solely of the Yi symbol, does it retain the capacity to guide viewers’ eye movements?

Another visual strategy concerns line connectedness and size [9]. For example, in a study depicting a man flying a kite, the rope directed participants’ gaze toward the kite. This reflects the same principle as a leading line, which guides attention toward a key element [14]. An open question, however, is whether this strategy is equally effective when applied to connect multiple symbols simultaneously. Size variation also affects eye gaze. Larger objects tend to receive viewing priority. Larger objects or areas with higher contrast often attract initial attention, guiding the viewer’s gaze through the composition. This may need to be tested with objects of equal size to determine whether larger objects can indeed lead the eye path.

Despite the cultural significance of Yi graphic symbols, current layout guidelines [13] often fail to account for their unique visual and semantic properties, leading to suboptimal gaze sequences and attention distribution. These optima fail to fully account for the visual and semantic properties of Yi symbols. Visually, Yi symbols are logographic and syllabic, featuring complex, non-linear shapes and artistic arrangements, unlike the simple hierarchies (e.g., text, images) targeted by Western design standards. Semantically, Yi symbols carry cultural meanings tied to their shape and position, which the optima, designed for universal readability, do not address. This mismatch motivates our study to explore context-specific strategies.

General studies on visual strategies (e.g., size, lines) show promise for gaze manipulation [9], but their application to Yi symbols remains underexplored, limiting their effectiveness in multimedia and interface design. To address this gap, this study tests four visual strategies color brightness, layering, line guidance, and size variation to optimize gaze sequences and attention. This experiment involves 34 Thai participants unfamiliar with Yi symbol meanings, ensuring a focus on universal visual perception. The primary objective is to validate visual strategies in Yi graphic symbol design by analyzing gaze sequences and attention patterns using eye-tracking. Specific objectives include assessing gaze alignment with intended sequences, identifying attention-grabbing components, and providing recommendations for culturally sensitive designs that enhance communication in multimedia applications.

## 2. Materials and Methods

### 2.1. Participant and Tasks

This study involved 34 Thai participants (18 males, 16 females; age range 20–57 years, M = 36.85, SD = 12.26), with demographic data collected to control for confounding variables and enable group analyses. Thai participants were selected to ensure cultural and perceptual consistency, as familiarity with Yi graphic symbols could introduce confounding factors like prior symbolic knowledge or cultural associations. By focusing on participants unfamiliar with Yi symbol meanings, the study isolated the effects of visual design features (color brightness, layering, line guidance, size variation) on gaze sequences and attention, independent of linguistic or cultural comprehension.

Each participant completed 8 randomized trials across four visual design feature manipulations (color brightness, layering, line guidance, size variation) in a within-subjects design, yielding 272 trials for analysis. The sample size was deemed sufficient based on power analysis (targeting 80% power to detect medium effect sizes, d = 0.5, with α = 0.05) and consistent with similar eye-tracking studies [7]. Trials were randomized using a Latin square design to balance the presentation order of the four visual strategies across 8 unique sequences, ensuring that each condition appeared equally often in each position. This randomization was implemented in SMI Experiment Suite 360 software (version 2.2), which generated a unique trial order for each participant to minimize order effects such as fatigue or learning. To further reduce order effects, participants were given a 30 s rest period after every four trials, and a practice session with five non-recorded trials was conducted to familiarize them with the task and reduce initial adaptation biases. Stimuli were presented for 450 ms following a 400 ms fixation cross, consistent across trials. Additionally, the first trial of each block was excluded to mitigate potential primacy effects, with preliminary analyses confirming that this exclusion did not significantly affect TTFF or dwell time (*p* > 0.05).

**Figure 1:**
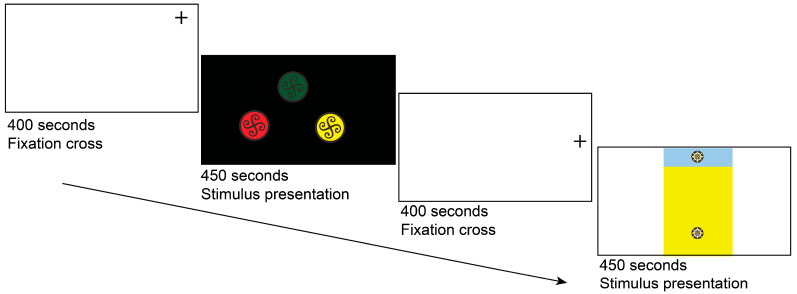
Eye-tracking trial sequence, with the fixation cross located outside the stimulus area.

Eye-tracking metrics, including Time to First Fixation (TTFF) for initial attentional guidance and dwell time for sustained attention. To prevent central fixation bias, a fixation cross was displayed outside the stimulus area for 400 ms (Figure 1), followed by a 450 ms stimulus presentation and a subsequent fixation cross. A 450 ms stimulus presentation was chosen to simulate brief exposure in real-world visual interfaces (e.g., mobile apps or packaging) and to minimize multiple fixations, focusing on initial attentional capture. This duration, while increasing TTFF variability, ensured ecological validity for rapid visual processing tasks.

### 2.2. Stimulus

**Figure 2:**
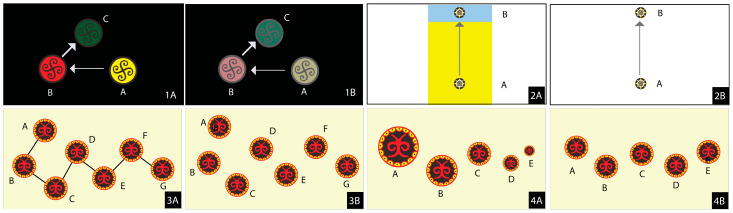
Stimuli for A/B testing: (**1A**) bright color, (**1B**) dull color; (**2A**) layered, (**2B**) non-layered; (**3A**) line-connected, (**3B**) non-connected; (**4A**) large-to-small size, (**4B**) equal size.

The stimuli were designed to evaluate four visual features—color brightness, layering, line guidance, and size variation on Yi graphic symbols using A/B testing (Figure 2). All stimuli excluded contextual elements (e.g., mountains, text) to focus on universal visual perception among Thai participants unfamiliar with Yi symbol meanings.

In the COLOR condition, three Yi graphic symbols were presented in bright colors (yellow, red, green; Figure 2(1A)), with yellow hypothesized to attract initial fixations due to its higher brightness, followed by red and green [9], compared to a control condition with the same symbols at equal brightness (dull colors; Figure 2(1B)). For the COLOR condition, the three Yi cultural colors (Yi Yellow, Yi Red, Yi Green) were sampled from traditional Yi symbol archives rather than standardized RGB primaries, to preserve cultural authenticity. When presented on the calibrated Dell 24” monitor (1920 × 1080, brightness 80%), their approximate luminance values were Yi Yellow ≈ 230 cd/m^2^, Yi Red ≈ 78 cd/m^2^, and Yi Green ≈ 145 cd/m^2^. The dull versions were created by reducing each Yi color sample to 30% of its original brightness while maintaining hue consistency, thereby providing a clear contrast between bright and dull conditions.

In this study, layering refers to a hierarchical arrangement of Yi graphic symbols by size, color, or position to guide the viewer’s gaze sequence, leveraging perceptual principles where larger or brighter elements attract initial attention, followed by smaller or background elements [12]. In the treatment condition (Figure 2(2A)), two symbols were arranged with a larger yellow symbol at the bottom (foreground) and a smaller blue symbol at the top (background), using a 1:6 size ratio, hypothesizing a bottom-to-top gaze sequence. The control condition (Figure 2(2B)) featured two symbols at the bottom and top without a size or color hierarchy (equal size, neutral colors) to serve as a baseline. Gaze sequence alignment was measured using Levenshtein distance and similarity ratio (Section 3.1), with Areas of Interest (AOIs) defined for the bottom and top symbols.

Line guidance involves connecting multiple Yi graphic symbols with a continuous line to direct the gaze sequence along a predetermined path, exploiting the tendency to follow linear cues [9]. In the treatment condition (Figure 2(3A)), six symbols were linked by a continuous line from left to right, hypothesizing a left-to-right gaze sequence. The control condition (Figure 2(3B)) placed six symbols in the same positions without connecting lines as a baseline. Sequence alignment was assessed with Levenshtein distance and similarity ratio, using AOIs for each symbol along the line.

In the SIZE condition, eight circular Yi graphic symbols were arranged from left to right in decreasing size (large-to-small; Figure 2(4A)), hypothesized to attract initial fixations to larger symbols before smaller ones [9], compared to a control condition with the symbols in the same positions but with equal size (Figure 2(4B)).

### 2.3. Apparatus

Eye movements were tracked using the SMI iView X RED4 system (version 2.2) with a 120 Hz sampling rate. Calibration was performed binocularly using a standard 9-point grid, with an acceptable error threshold of ≤0.5° of visual angle. Participants who exceeded this threshold were recalibrated until the criteria were met, ensuring high spatial accuracy in fixation-based analyses. Areas of Interest (AOIs) were analyzed using BeGaze 2 (version 2.3).

### 2.4. Data Analysis

The Levenshtein distance is a metric that measures the minimum number of single-character edits insertions, deletions, or substitutions required to transform one string into another, quantifying the difference between two sequences. For example, to transform the string “kitten” into “sitting”, the process involves three edits: substituting “k” with “s”, substituting “e” with “i”, and inserting “g” at the end, resulting in a Levenshtein distance of 3. This metric is widely used to assess the dissimilarity between sequences, such as gaze sequences in eye-tracking studies.

In our analysis, the Levenshtein distance was applied to compare gaze sequences of fixations across AOIs (e.g., B, A, C) generated by participants viewing Yi symbols under different visual strategies (e.g., color, layering). Lower Levenshtein distances indicate more similar gaze sequences, suggesting that a visual strategy (e.g., bright red) effectively guided gaze in a consistent pattern. The similarity ratio, derived from the Levenshtein distance, is defined as 1 minus the normalized Levenshtein distance divided by the length of the longer sequence, providing a value between 0 and 1 where higher values indicate greater similarity. For instance, if the Levenshtein distance between two gaze sequences is 2 and the longer sequence has 10 fixations, the similarity ratio would be 1 − (2/10) = 0.8, reflecting an 80% similarity.

This similarity ratio relates directly to our findings by quantifying how well visual strategies align gaze behavior. For example, a higher similarity ratio for bright red conditions (as noted in the results) indicates that color contrast reduced gaze sequence variability, supporting its role in directing initial attention. The metric complements TTFF and dwell time data, offering a holistic view of gaze guidance across the four strategies. The methodology has been updated to include this definition and example, ensuring clarity, and the results section now explicitly ties the similarity ratio to Levenshtein distance trends observed in the raw data.

Time to First Fixation (TTFF), defined as the latency from the onset of the stimulus (following a 400 ms fixation cross) to the start of the first fixation within an Area of Interest (AOI) lasting at least 100 ms during the 450 ms presentation window, was measured in milliseconds and served as a proxy for initial attentional capture due to its sensitivity to visual saliency, such as color or contrast. A fixation was identified using a velocity-based algorithm in BeGaze software (version 2.3), with eye movement velocity below 30°/s and a 1.5° dispersion threshold for 100 ms, ensuring stable gaze periods. TTFF was calculated automatically by BeGaze, timestamping the stimulus onset and the start of the first qualifying fixation within an AOI. Lower TTFF values typically indicate faster attention capture, often influenced by visual salience or cultural significance of Yi symbols, while higher values may suggest delayed orientation due to reduced prominence or competing design elements.

Dwell time was calculated as the total duration of fixations, each lasting at least 100 ms, within an AOI during the 450 ms stimulus presentation, reflecting sustained attention and engagement with design elements. These metrics were chosen because TTFF indicates how quickly visual strategies (e.g., color brightness) attract attention, while dwell time reveals how long attention is maintained, critical for effective visual communication in Yi symbol designs. Paired samples *t*-tests (two-tailed, α = 0.05) compared Levenshtein distance, similarity ratio, and TTFF between treatment and control stimuli across conditions. Effect sizes were computed as Cohen’s d (d = M_diff/SD_diff) to quantify the magnitude of differences in gaze behavior and attention.

## 3. Results

This section presents the results of the eye-tracking experiment, focusing on gaze sequence analysis and Area of Interest (AOI) metrics. Gaze sequence analysis evaluated alignment between observed and intended gaze sequences using Levenshtein distance and similarity ratio, while AOI metrics assessed attention through Time to First Fixation (TTFF) and dwell time across four design conditions: color brightness, layering, line guidance, and size variation.

### 3.1. Gaze Sequence Analysis

In the color condition, bright Yi graphic symbols show significantly better alignment with intended gaze sequences than dull symbols, with lower Levenshtein distance, t (33) = −2.86, *p* = 0.007, and higher similarity ratio, *t* (33) = 2.50, *p* = 0.017. Similarly, layered symbols demonstrate stronger gaze sequence guidance than non-layered symbols, with lower Levenshtein distance, *t* (33) = −2.81, *p* = 0.008, and higher similarity ratio, t (33) = 2.57, *p* = 0.015. In the line condition, connected symbols align better with intended gaze sequences than non-connected symbols, with lower Levenshtein distance, *t* (33) = −2.56, *p* = 0.015, and higher similarity ratio, *t* (33) = 2.56, *p* = 0.015. In contrast, the size condition show no significant differences in Levenshtein distance, *t* (33) = −1.38, *p* = 0.178, or similarity ratio, *t* (33) = 1.40, *p* = 0.170, indicating size variation did not significantly influence gaze sequence alignment (Table 1). Levenshtein distance and similarity ratio results are provided in the Supplementary Material.

### 3.2. AOI Metrics

Paired-samples *t*-tests compare TTFF between Bright and Dull features across AOIs (*n* = 34). For AOI B (Red), Bright symbols significantly reduce TTFF (M = 187.21 ms, SD = 151.80) compared to Dull (M = 308.31 ms, SD = 363.59), *t* (33) = −2.71, *p* = 0.011, d = −0.46. No significant differences were observed for AOI C (Green; *p* = 0.524, d = −0.11) or AOI A (Yellow; *p* = 0.973, d = 0.006).

Dwell Time analyses revealed non-significant trends toward longer fixation durations for Dull compared to Bright in AOI B (929.04 vs. 789.56 ms) and AOI A (767.46 vs. 729.56 ms), with minimal differences in AOI C (1085.29 vs. 1070.00 ms). Table 2 summarizes these findings.

With Bonferroni correction (α = 0.025 for two AOIs), paired-samples *t*-tests showed no significant TTFF differences between Layered and Non-layered features (*n* = 34). For AOI B (Top), TTFF was shorter in the Layered condition (M = 232.85 ms, SD = 196.33) than in the non-layered condition (M = 314.17 ms, SD = 311.17), but this difference was not significant, *t* (33) = −1.61, *p* = 0.117, d = −0.28. For AOI A (Bottom), TTFF values were nearly identical (Layered: M = 377.36 ms, SD = 547.26; Non-layered: M = 363.59 ms, SD = 259.04), *t* (33) = −0.02, *p* = 0.987, d = −0.003.

Dwell Time comparisons also revealed no significant differences (AOI B: *p* = 0.785, d = −0.05; AOI A: *p* = 0.916, d = −0.02), with variability remaining high (SD > 1000 ms). Table 3 provides descriptive statistics and test results.

**Figure 3:**
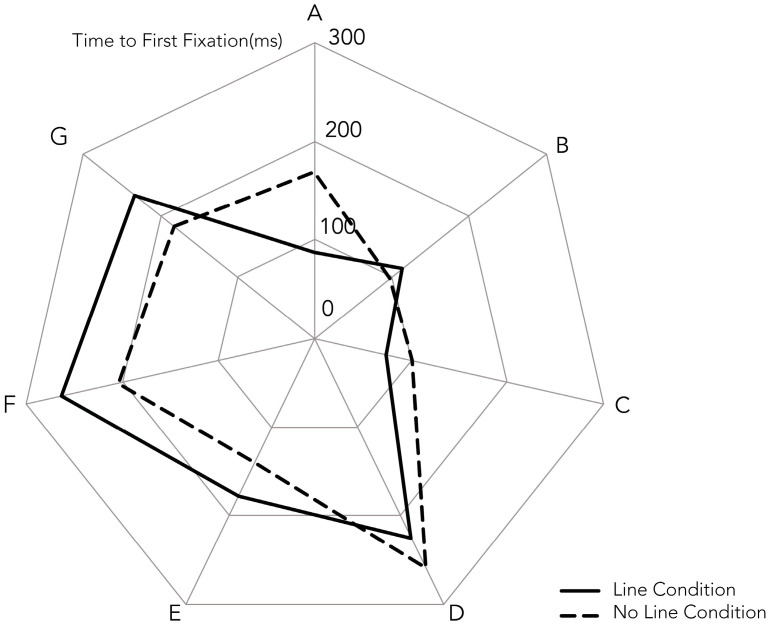
Mean Time to First Fixation (TTFF) for line and no-line conditions across seven Areas of Interest (AOIs A to G).

Time to first fixation (TTFF) is compared between the line and no-line conditions across seven AOIs (Figure 3). Overall, the effects of line cues on TTFF are small. For AOIs G, F, and E, TTFF is slightly longer in the line condition than the no-line condition, but these differences are nonsignificant (e.g., AOI F: M = 263.84, SD = 201.66 vs. M = 204.59, SD = 214.80), *t* (33) = 1.22, *p* = 0.230, d = 0.21. By contrast, AOIs D, C, and B show slightly faster TTFF in the no-line condition, but again the effects are trivial and nonsignificant (e.g., AOI C: M = 74.30, SD = 132.15 vs. M = 101.79, SD = 156.06), *t* (33) = −0.96, *p* = 0.344, d = −0.16. The only significant effect is observed at AOI A, where participants fixated faster in the line condition (M = 87.17, SD = 137.97) compared to the no-line condition (M = 168.49, SD = 238.35), *t* (33) = −2.16, *p* = 0.038, d = −0.37, indicating a small-to-medium effect. Thus, AOI A was the primary area where line cues successfully guided initial attention.

Dwell time, the total duration of fixations in each area of interest (AOI), differed from time to first fixation (TTFF). Paired-samples *t*-tests (*n* = 34, α = 0.05, two-tailed) compared dwell times between line and no-line conditions across seven AOIs (A–G). For AOI A, dwell time was significantly shorter in the line condition (M = 105.89 ms, SD = 169.88) than in the no-line condition (M = 250.99 ms, SD = 309.36), *t* (33) = −2.95, *p* = 0.006, indicating that line cues facilitated rapid disengagement after capturing attention. For AOI F, dwell time was longer in the line condition (M = 600.25 ms, SD = 571.88) than in the no-line condition (M = 377.19 ms, SD = 454.65), *t* (33) = 2.18, *p* = 0.036 but this difference was not significant after Bonferroni correction. A similar trend was observed for AOI G. No significant differences emerged for AOIs B, C, D, or E. Thus, line cues primarily reduced dwell time at the sequence’s start (AOI A), supporting their role in guiding attention, with variable effects in later AOIs.

**Figure 4:**
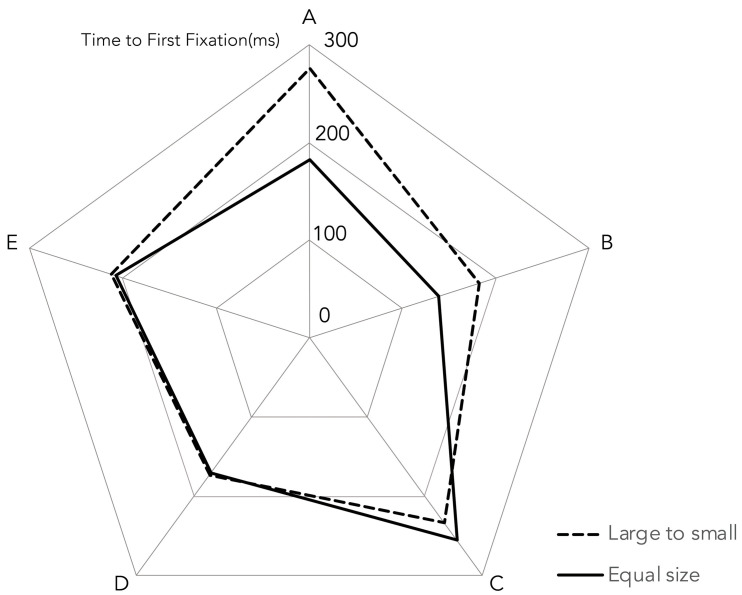
Mean Time to First Fixation (TTFF) for large-to-small and equal size conditions across seven Areas of Interest (AOIs A to E).

Time to First Fixation (TTFF) does not significantly differ between the large-to-small and equal-size conditions across AOIs (Figure 4). For example, at AOI E, TTFF was nearly identical between conditions (M = 211.77 ms vs. 207.68 ms), *t* (33) = 0.08, *p* = 0.940, d = 0.01. Similarly, nonsignificant effects are observed at AOIs D and C (ds ≤ 0.11). A trend toward faster fixation in the large-to-small condition is observed at AOI B (M = 182.55 ms vs. 138.06 ms), *t* (33) = 1.70, *p* = 0.098, d = 0.29, although this does not reach corrected significance. Likewise, AOI A showed a significant difference, with longer TTFF in the large-to-small condition (M = 276.72 ms) compared to equal-size (M = 182.54 ms), *t* (33) = 1.98, *p* = 0.056, d = 0.34.

Dwell time, the total duration of fixations in each area of interest (AOI), was compared between large-to-small and equal size conditions across five AOIs (A–E). Paired-samples *t*-tests (*n* = 34, α = 0.05, two-tailed) revealed a trend for longer dwell time at AOI A in the large-to-small condition (M = 552.85 ms, SD = 526.21) compared to the equal size condition (M = 275.56 ms, SD = 295.54), *t* (33) = 2.68, *p* = 0.011, though this was not significant. A trend toward longer dwell times in the large-to-small condition was observed at AOI B (large: M = 510.16 ms, SD = 427.72; equal: M = 354.53 ms, SD = 391.40), *t* (33) = 1.71, *p* = 0.096, and in the equal size condition at AOI C (large: *M* = 842.43 ms, SD = 685.18; equal: M = 1203.40 ms, SD = 1030.46), *t* (33) = −1.99, *p* = 0.055. No significant differences emerged for AOIs D or E (*ps* > 0.05), with high variability in responses (e.g., SD = 1030.46 for AOI C, equal size). These findings suggest that larger sizes at the sequence’s start (AOI A) may sustain attention longer, with variable effects in later AOIs.

## 4. Discussion

This eye-tracking study explored how color brightness, layering, line guidance, and size variation shape attention in Yi graphic symbols among Thai participants. By examining gaze sequence alignment and attention metrics TTFF and dwell time), the findings illuminate the distinct roles of these visual strategies, offering insights for designing culturally unfamiliar visual displays and advancing theories of visual attention.

Color brightness effectively guided participants’ gaze along intended sequences in Yi graphic symbols, ensuring more accurate alignment compared to dull colors, consistent with research on visual salience [15]. The effect varied by hue: vibrant red symbols captured attention faster, likely due to their high perceptual and cultural salience, while yellow and green showed minimal impact on initial fixations [16,17,18]. Sustained attention, as measured by dwell time, showed no consistent differences across hues, suggesting that brightness primarily influences initial gaze capture rather than prolonged engagement [19]. Designers should thus prioritize hue, brightness, and task relevance when using color to guide attention in culturally unfamiliar displays like Yi symbols.

Layered designs, creating a sense of depth, enhanced gaze alignment, supporting the role of hierarchical spatial arrangements in facilitating perceptual flow. These designs guided directional movement but had little effect on sustaining engagement, suggesting they function best when combined with other cues like color or lines. Line connections served as explicit navigational guides, anchoring attention at the sequence’s start and enabling efficient progression by prompting quicker disengagement, consistent with their role as perceptual continuities. Their influence diminished in later areas, indicating that lines are most effective at entry points.

In contrast, size variation showed little impact on guiding gaze sequences, with large-to-small arrangements offering no clear advantage over equal-sized symbols. However, larger sizes at the sequence’s start tended to sustain attention longer, promoting deeper engagement with initial elements before viewers moved forward. This effect was inconsistent in later areas, likely because the intricate design of Yi symbols overshadows size-based cues, requiring more explicit guidance like color or lines [20]. For Thai participants, the cognitive effort needed to process unfamiliar symbols may reduce size’s effectiveness as a standalone cue, highlighting the interplay between visual hierarchy and cultural familiarity.

Initial attention capture further distinguished the strategies. Bright red symbols and lines drew gaze faster, acting as direct attentional anchors, while layering and size variation had minimal impact, indicating that explicit cues are critical for overcoming initial processing challenges in complex Yi symbols. Dwell time patterns showed lines enabling efficient progression, larger sizes encouraging longer engagement at the start, and color and layering lacking consistent effects, with high variability reflecting diverse participant responses, possibly due to differences in processing abstract symbols [18].

These findings inform Yi graphic symbol design for applications like educational materials or cultural exhibits. Bright colors and line connections are robust tools for guiding attention, ensuring efficient navigation for audiences unfamiliar with the symbols. Layering supports sequence following but may require integration with other cues for maximum impact. Size variation, while less effective for guiding gaze, enhances engagement at key points, suggesting a complementary role. For instance, starting with a larger, brightly colored symbol connected by lines could optimize both attention capture and sequence navigation.

Theoretically, the results extend visual attention research by showing how cue effectiveness varies with stimulus complexity and cultural context. While saliency models predict universal guidance from bright colors and lines, the limited impact of size variation underscores how cognitive and cultural factors, such as unfamiliarity with Yi symbols, modulate these effects. High dwell time variability suggests that individual differences, such as visual processing speed or exposure to abstract symbols, play a significant role, warranting further exploration in cross-cultural settings.

Limitations include the study’s focus on Thai participants, which may limit generalizability to other cultural groups with different perceptual or aesthetic traditions. The restricted number of stimuli and emphasis on bottom-up visual cues also leave questions about top-down influences, such as task goals or symbol familiarity. Future research could test these strategies in diverse populations and naturalistic settings, exploring combined cues (e.g., color with lines) or dynamic gaze analyses to optimize attention guidance in Yi symbols and similar complex displays. These insights pave the way for designing visually engaging, culturally accessible graphic systems.

Table 4 summarizes the comparative effectiveness of the four visual strategies across gaze sequence alignment, initial attentional capture, and sustained engagement. The results show that color brightness and layering primarily facilitated gaze flow, while line guidance proved consistently effective across all measures. In contrast, size variation played only a limited role, influencing dwell time rather than the initiation or direction of gaze. To aid interpretation for design practitioners, Table 4 translates the experimental findings into a simplified effectiveness rating, highlighting which visual strategies are most effective for guiding, capturing, and sustaining attention in Yi symbol layouts.

## 5. Conclusions

The findings of this study demonstrate that visual strategies have differential effects on gaze behavior in Yi graphic layouts. Color brightness, layering, and line guidance effectively guided initial attention, as indicated by shorter gaze sequence distances and higher similarity, with bright colors and connected or layered elements drawing attention more efficiently. In contrast, size variation did not significantly affect the timing of first fixations but strongly influenced dwell time, suggesting that larger elements sustain attention once noticed. Overall, these results highlight that color, layering, and line cues are effective for directing gaze, while size primarily supports prolonged engagement, providing practical insights for optimizing the design of culturally meaningful Yi graphics.

## Figures and Tables

**Table 1:** Paired-Samples *t*-Tests for Levenshtein Distance and Similarity Ratio.

Comparison	Visual Feature	Condition	M	SD	*t* (33)	*p*
Color-Distance	Color	Bright	1.32	0.94	−2.86	0.007 *
		Dull	1.85	0.66		
Color-Similarity	Color	Bright	56.71	30.69	2.50	0.017 *
		Dull	42.24	20.58		
Layer-Distance	Layer	Layered	0.65	0.85	−2.81	0.008 *
		No Layered	1.21	0.91		
Layer-Similarity	Layer	Layered	64.71	43.57	2.57	0.015 *
		No Layered	39.71	45.69		
Line-Distance	Line	Connected	4.65	1.32	2.56	0.015 *
		Non-connected	3.91	1.75		
Line-Similarity	Line	Connected	44.15	24.94	−2.56	0.015 *
		Non-connected	33.82	19.05		
Size-Distance	Size	Large-to-Small	2.24	1.46	−1.38	0.178
		Equal Size	2.59	1.33		
Size-Similarity	Size	Large to Small	56.09	28.19	1.40	0.170
		Equal Size	48.94	25.90		

Note: All tests are two-tailed, α = 0.05. * *p* < 0.05.

**Table 2:** Time to First Fixation (TTFF) and Dwell Time for Color Brightness Condition.

AOI	Condition	TTFF M (SD)	*t* (33), *p*, d	Dwell Times M (SD)	*t* (33), *p*, d
Green (C)	Bright	241.64 (159)	−0.64, 0.524,−0.28	1085.29 (726)	0.14, 0.892, 0.05
	Dull	261.51 (203)		1070 (738)	
Yellow (A)	Bright	253.91 (176)	0.03, 0.973, 0.006	729.56 (465)	−0.36, 0.724, −0.12
	Dull	252.64 (140)		767.46 (498)	
Red (B)	Bright	187.21 (151)	−2.71, 0.011 *, −0.46	789.56 (672)	−1.16, 0.253, −0.20
	Dull	308.31 (363)		929.04 (749)	

Note. Significant effect of brightness observed only for AOI B–Red (shorter TTFF for Bright vs. Dull, * *p* < 0.05). No significant differences in dwell time across AOIs.

**Table 3:** Time to First Fixation (TTFF) and Dwell Time for Layered Condition (Layered vs. Non-layered).

AOI	Condition	TTFF M (SD)	*t* (33), *p,* d	Dwell Times M (SD)	*t* (33), *p,* d
Top (B)	Layered	232.85 (196)	−1.61, 0.117, −0.28	1019.73 (1041)	−0.28, 0.785, 0.005
	Non layered	314.17, 311		1050.15 (875)	
Bottom (A)	Layered	377.36 (547)	−0.02, 0.987, −0.003	2027.74 (1095)	−0.11, 0.916, −0.02
	Non layered	363.59 (259)		2041.8 (1039)	

Note. No significant TTFF or dwell time differences were observed between Layered and Non-Layered conditions. Variability in dwell times was high across AOIs.

**Table 4:** Effective of Visual Strategies for Guiding Attention to Yi Symbols.

Visual Strategy	Gaze Sequence	Capture Attention (TTFF)	Sustain Attention (Dwell Time)	Core Insight
Color Brightness	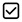	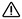	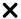	Excellent for guiding gaze, but only bright red significantly captured initial attention. Brightness primarily influences initial capture, not sustained engagement.
Layering	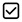	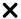	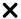	Helps guide overall flow and directional movement, but doesn’t capture or hold attention on its own. Works best when combined with other cues.
Line guidance	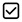	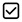	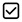	Powerful for anchoring attention at the start and making navigation more efficient. It captured attention faster and then prompted quicker disengagement from the first symbol.
Size variation	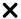	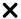	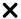	Not effective for guiding gaze sequence, but larger symbols at the start tended to hold attention longer. The complexity of the unfamiliar Yi symbols likely overshadowed size cues.

Note. 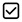 Effective: Statistically significant positive effect, 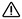 Partially Effective: Mixed or trend-level effects, 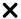 Not Effective: No statistically significant effect found.

## Data Availability

The data that support the findings of this study are openly available in Zenodo at https://doi.org/10.5281/zenodo.17358646 (accessed on 15 October 2025).

## Supplementary Materials

The following supporting information can be downloaded at: https://www.mdpi.com/article/10.3390/jemr18050057/s1, Levenshtein distance and similarity ratio results are provided in the Supplementary Material.

## Abbreviations

The following abbreviations are used in this manuscript:

AOI	Area of Interest. Specific region in a visual stimulus analyzed for gaze behavior.
d	Cohen’s d. Effect size for paired-samples, measuring the magnitude of the difference.
*p*	*p*-value. Probability indicating statistical significance of a result.
SD	Standard Deviation. Measure of variability or dispersion in the data.
TTFF	Time to First Fixation. Time elapsed from stimulus onset until the first fixation on an AOI.
